# The need for a worldwide consensus for cell line authentication: Experience implementing a mandatory requirement at the *International Journal of Cancer*

**DOI:** 10.1371/journal.pbio.2001438

**Published:** 2017-04-17

**Authors:** Norbert E. Fusenig, Amanda Capes-Davis, Franca Bianchini, Sherryl Sundell, Peter Lichter

**Affiliations:** 1 German Cancer Research Center (DKFZ), Heidelberg, Germany; 2 CellBank Australia, Children's Medical Research Institute, University of Sydney, Westmead, New South Wales, Australia; 3 German Cancer Research Center (DKFZ), *International Journal of Cancer*, Heidelberg, Germany; 4 Division of Molecular Genetics and German Cancer Consortium (DKTK), German Cancer Research Center (DKFZ), Heidelberg, Germany

## Abstract

Cell lines are used in life science research worldwide as biological surrogates. All cell lines are subject to major limitations when used as research tools, including (i) cross-contamination with other cells cultured in the same laboratory environment and (ii) evolution in vitro that renders a given cell line inappropriate as a surrogate for a specific biological hypothesis. There is ample evidence that cross-contamination or phenotypic drift of cells in culture can generate irreproducible or misleading data. A small number of scientific journals—the *International Journal of Cancer* being at the forefront—and funding agencies have recently moved forward to ask for obligatory cell line authentication data. The history of implementing such rules by the *International Journal of Cancer* exemplifies the difficulties encountered when installing mandatory quality measures in life sciences.

## Introduction

Cell lines are considered to represent cell functions in a given tissue of an organism and, therefore, have become invaluable tools for biomedical research. To maintain the role of biological surrogates, cell lines require constant quality control and assurance of their identity throughout their lifetime in vitro. Particularly if derived from tumor tissues, cells change over time in culture, both phenotypically and genetically. An even more disastrous feature is the cross-contamination that frequently occurs when cells from another culture are introduced by accident, resulting in replacement by a contaminant that has been propagated in the same laboratory or biosafety cabinet and is better adapted and faster growing than the original, authentic culture. The end result of cross-contamination is a cell line that no longer corresponds to the original, authentic culture. In addition to cross-contamination, cell line misidentification can arise from incorrect labeling at the source. Misidentification of cell lines can happen in every laboratory, and without constant and vigorous control of authenticity, it often remains undetected.

Postgenomic research highlights continuously more detailed genetic and epigenetic differences between tumor entities originating from the same organ and even within the heterogeneous populations of a single tumor [1,2]. Thus, in the future, even more care has to be taken in selecting the correct cell line—including constant quality control—as well as in carefully interpreting the derived data. As tumor cell lines often represent the first-line test objects for drug development, many biopharmaceutical companies have taken measures to check their cell lines regularly and employ cell line repositories, resulting in a low frequency of cell line misidentification [3,4].

We would make far more progress if cancer research labs and centers would follow a similar policy. The German Cancer Research Center has a mandatory requirement that all manuscripts to be submitted must authenticate the cell lines used in the experiments prior to submission. A similar policy is in place at the MD Anderson Cancer Center [5]. Some laboratories have policies in place, for example, requiring authentication whenever a cell line enters or leaves a laboratory [6].

Problems with misidentification and cross-contaminations have been regularly brought to light in the cell biology community but have only resulted in very little change. Indeed, worldwide, between 14 and 46% of the most commonly used cell lines are believed to be incorrectly designated [7–11]. Things started to slowly change after the turn of the millennium. New testing methods became available, with John Masters leading an initiative to assess the technique of short tandem repeat (STR) profiling for cell line authentication [12]. Many of the major cell banks took the initiative to inform their clients and withdraw false cell lines from their catalogues. In this initiative, the German Collection of Microorganisms and Cell Cultures (Leibniz Institut-DSMZ) has been a pioneer and is still a major actor in the process.

A number of genotype-based testing methods are now available, with a recent increase in molecular approaches such as STR profiling and SNP analysis [13]. Cell banks and research groups share their datasets and publish on this topic (e.g., [14–18]), and standardized DNA databases of STR profiles from human cell lines are publicly available [19]. Scientists based at Genentech recently published a comprehensive collection of 2,787 STR profiles and 1,020 SNP profiles for many commonly used human cell lines, brought together from publicly available datasets and additional testing data [13]. The American Type Culture Collection (ATCC) Standard Development Organization established a working group of international experts to develop a written standard for human cell line authentication [20]. The standard and associated publications have reignited the warning to the scientific community that proper mandatory regulations for authentication need to be in place. This warning is particularly important for editors of scientific journals and representatives of funding organizations [21]. The large number of ~500 cell lines affected means that it is impossible for authors or journal editors to be aware of all instances.

### Usage of misidentified cell lines in biomedical journals

Journals are key stakeholders in resolving the problem of misidentified cell lines [22]. For more than a decade, the *International Journal of Cancer* (*IJC*) has been publishing articles to increase awareness and understanding of misidentified cell lines and to provide resources for authentication testing [8,9,19]. Some journal editors have published on the need for authentication testing and have incorporated authentication testing as a requirement for authors, including *BioTechniques* [23,24], *Clinical Cancer Research* [25], *Nature* [26], and the *IJC* [27]. Prominent journals that currently incorporate authentication testing into their author guidelines are listed in Table 1. This list may not be exhaustive, however, as there is no means of alerting the scientific community to those journals that require testing and those that do not.

**Table 1:** Journals that include instructions or requirements for cell line authentication in their author guidelines [28].

Publisher and/or Journal Title	Required	Encouraged
American Association for Cancer Research journals (8)		X
BioMed Central journals (200+)		X
Endocrine Society journals (5)	X	
*Nature* journals (approximately 150)	X	
Society for Endocrinology journals (3)	X	
*BioTechniques*		X
*Carcinogenesis*	X	
*Cell Biochemistry and Biophysics*	X	
*Cell Biology International*	X	
*International Journal of Cancer*	X	
*Investigative Ophthalmology & Visual Science*		X
*In Vitro Cellular & Developmental Biology—Animal*	X	
*Journal of Molecular Biology*		X
*Journal of the National Cancer Institute*	X	
*Molecular Vision*	X	
*Neuro-Oncology*		X
*PLOS ONE*		X

**Fig 1:**
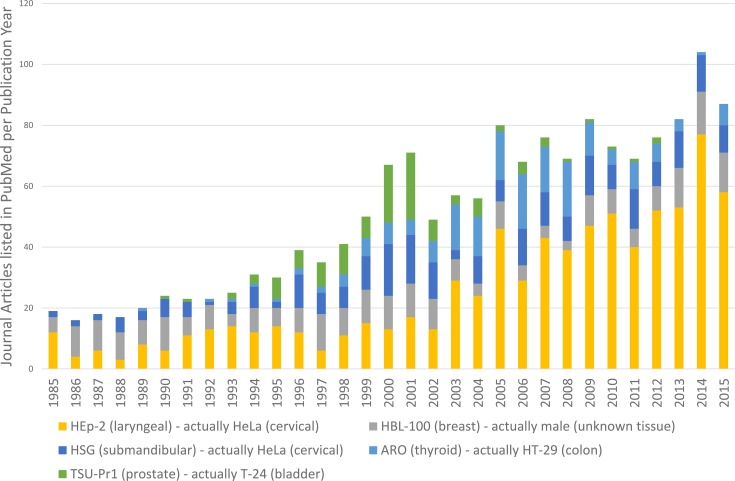
Usage of five misidentified cell lines in the scientific literature as shown by PubMed searches for cell line name and incorrect tissue identity.

Despite numerous publications by the *IJC* and other journals [14–18], authors have continued to submit work that used misidentified cell lines. Examples may help to explain the magnitude of the problem. Fig 1 examines usage of five known misidentified cell lines: HEp-2, HBL-100, HSG, ARO, and TSU-Pr1. All were originally thought to come from a specific tissue—for example, HEp-2 was originally established from a male with laryngeal carcinoma [29]. All are now known to be misidentified, with the contaminating cell line arising from a different tissue—for example, Gartler demonstrated that HEp-2 is actually HeLa, arising from a female with cervical carcinoma [30].

Fig 1 was compiled from PubMed searches looking at usage of these five misidentified cell lines across multiple journals. Each search was set up to detect the cell line name in association with the incorrect tissue—for example, “HEp-2” and “laryngeal”—in an article’s title or abstract. HEp-2 is widely used in publications that study laryngeal cancer, despite the fact that it was shown to be a HeLa derivative by Gartler [30]. Our HEp-2 data are also consistent with work by Christopher Korch, who has identified 5,789 articles that use HEp-2 inappropriately [31]. Other cell lines may peak and then decline in use—for example, usage of the TSU-Pr1 cell line declined after it was shown to be T-24, from bladder carcinoma, in 2001 [32]. This is consistent with previous analysis of ECV-304, another misidentified cell line, for which negative publicity was associated with a sharp decline in usage [33]. All five examples have been used in multiple journals, including the *IJC*. Looking at search results from these five cell lines, it is obvious that overall usage is steadily increasing.

### Stepwise measures to implement cell line authentication: The *IJC* experience

Roland Nardone’s 2007 call to action concerning the problem of cell line contamination in biomedical research [34] prompted considerable discussion among the editors of the *IJC* and dominated the agenda of the weekly editors’ meetings for some time. As outlined in Nardone’s letter, many factors were weighed in our discussion, including the corruption of biomedical research in general as well as the matters of educating researchers and of compliance. For the *IJC* editors, how such a measure would affect submissions and the journal’s reputation represented important considerations. Ultimately, we decided that the most effective way of handling the matter would be to establish a consortium of journals requiring proof of the authenticity of cells at manuscript submission. With this goal in mind, the *IJC* contacted several high-impact general, cell biology, and cancer research journals in October of 2007 and proposed this idea. At a later point in 2009, the journal solicited some granting agencies to adopt a policy that would require mandatory DNA profiling as a prerequisite for receiving funding.

**Fig 2:**
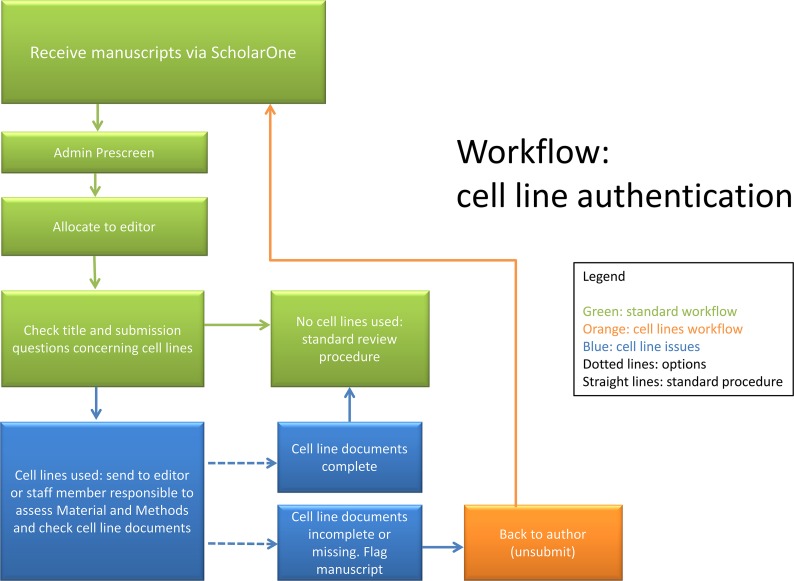
Workflow at the *IJC*, including checking of cell line documents.

Unfortunately, the publishing community basically did not respond to this call to action. In the few answers the editors received, journals and granting organizations acknowledged the problem but saw the responsibility for control in the hands of reviewers and the scientists themselves. In 2010, since no joint initiative with leading journals and granting organizations could be established, the *IJC* decided to “go it alone” [27] and after a short pilot period during which authors were encouraged to authenticate their lines, we made it obligatory that authors submitting to the journal provide DNA-based certification of the identity of the cell lines used in their work. As a simple aid, we recommended that authors first check the published list of known cross-contaminated cell lines and then perform authentication. Implementing such a procedure at the journal was not without difficulties and required a series of administrative and editorial measures, as summarized in Box 1. We also needed to expand our procedures for logging in manuscripts to include checking whether cell lines had been used in a submitted manuscript and whether the proper documentation had been provided (Fig 2). At submission, each paper was checked, and in order to monitor the history of the papers, we compiled and maintained a list in Excel of those manuscripts with cell line problems at submission. We verified whether cell lines were used and whether certificates were uploaded and fulfilled our criteria, which were as follows: cell lines had been purchased from a reliable source within the past four years as per date on purchase order or invoice, cell lines were profiled within the past four years at a cell bank or reliable service provider and showed at least 80% identity, or cell lines were authenticated in the author’s lab within the past four years with the profiling data provided and a form signed indicating that the results had been counterchecked against the standard database. If the documents were not valid, the paper was returned to the authors (“unsubmitted,” which here means editorial consideration was paused). In our Excel file, we also recorded when the paper was submitted again and documented the final decision on the paper. It is worth mentioning that in many cases the authors declared that they didn’t use cell lines in their study, although they did, implying that each paper had to be carefully checked to verify the answer given in the submission questions. The editors discussed whether we should consider a false declaration as fraud and thus as sufficient reason for the immediate rejection of the paper or merely a misinterpretation of the new procedure. We opted for this second option and gave the authors the chance to resubmit their paper with the appropriate documents.

Box 1. Implementation of the procedure at the IJC*

***STEP 1*. 2010—Cell line authentication is recommended.**
  - Author instructions amended to include this recommendation.
  - Authors informed on how and where they could have profiling done.
***STEP 2*. Starting from 2011—Cell line authentication, not older than four years, becomes necessary requirement for publication.**
  - Author instructions rewritten to reflect new stipulation regarding cell lines.
  - E-mail templates developed to address the new requirements.
  - Manuscript managing system (ScholarOne) updated to include new questions for authors about whether or not they authenticated their cell lines.
  - All manuscripts checked upon submission to verify whether human cell lines were used and whether corresponding files proving authentication were uploaded.
  - Papers flagged until a valid certificate was obtained.
  - Papers are monitored regarding the following: date of submission, title, author, country, resubmission or withdrawal, and final decision.
***STEP 3*. Starting from 2012—Papers not complying with requirement are unsubmitted.**
  - The authors use cell lines and do not provide the certificate.
  - The certificate provided does not fulfill the requirements (i.e., STR profile older than four years).
  - The authors declare “no cell lines used” but on inspecting the manuscript, it appears that cell lines were used.
***STEP 4*. Statistical analysis of data, including the following:**
  - Rates of returning the papers to the authors
  - Rates of resubmission by year
  - Rates of resubmission by continent
  - Rates of acceptance by year

*From poster submitted to the International Society of Managing and Technical Editors (ISMTE) 2012

In addition to the considerable time and cost involved in monitoring the process, these new requirements also had to be included in the author instructions, in the email template sent to the authors to ask for suitable certificates, and in the submission questions in the ScholarOne manuscript system. Once the procedure was in place, we had to describe it in detail for our internal office manual. Especially at the beginning, many researchers were reluctant to check their own cell lines (see Box 2). In particular, some only became aware of the new procedure during or after submitting their papers for publication, at which point it was too late to change data or findings if cell lines were indeed contaminated. Other scientists with fewer resources, as in the case of developing countries, couldn’t easily perform the necessary authentication, thus risking their chance of publication because of the new requirements.

**Box 2. Problems arising as a result of the new requirement that had to be dealt with on a “case-by-case” basis**

- Authors claimed that cell lines are a gift from another lab, have already been used in several publications, no previously validated DNA exists for comparison, or they believe that because lines are not in the list of cross-contaminated cell lines they should be ok.
- Authors only used further-modified cells or claimed that a cell line experiment is not really the main focus of the paper.
- Authors sent only data sheets from a cell bank and declared this as authentication.
- Authors sent data in illegible form, sent just the *IJC* form without further STR data for proof, or sent only raw data without a written statement of control and percentage of match.
- Authentication of cell lines was older than the four-year limit currently implemented by the *IJC* or cell lines were established more than four years ago but were cultured only for short periods from frozen samples.
- Authors wanted to have profiling done while paper was under review or only if they could be assured paper would be accepted.
- Authentication could not be performed because of a lack of resources or authors were not aware of guideline and were not willing to have lines profiled.

Considerable administrative time and cost needed to be invested as a consequence: we estimated approximately 240 hours of additional work over this three-year period to process papers that used cell lines (i.e., logging in the manuscript, checking the files, and entering the information into our Excel file and then returning the paper to the authors). The same amount of time per paper was needed again to log in the paper and update the Excel file if the paper was resubmitted. On top of that, editorial expertise and time were needed to check the actual documents, which vary for papers with good documentation and manuscripts that include numerous cell lines. In contrast to the administrative times, which in the meantime have evened out with experience, these editorial times still apply and still vary according to quality of documentation and number of cell lines used.

### Outcomes

**Fig 3:**
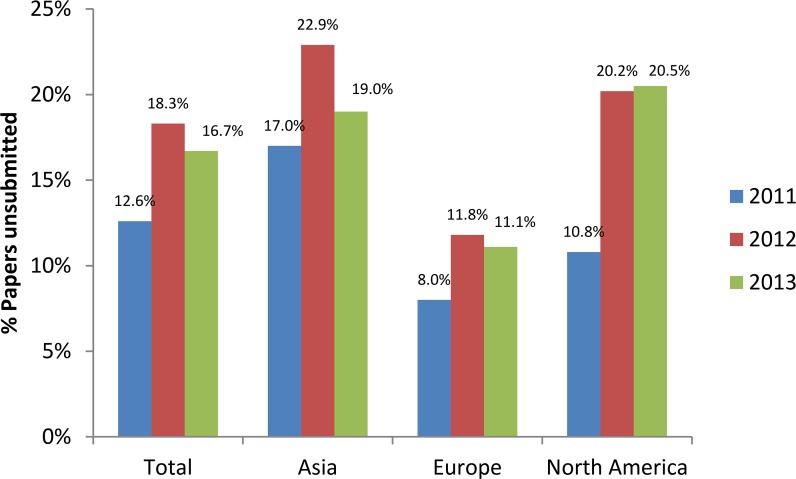
Percentages of papers unsubmitted for cell line issues, by continent and by year. Generated from S1 Data and S2 Data.

**Fig 4:**
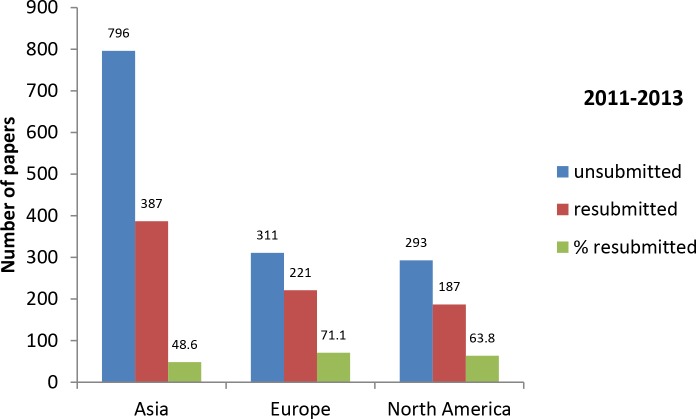
Papers unsubmitted for cell line issues and then resubmitted, by continent in 2011–2013. Generated from S1 Data.

After implementing the new requirements, we monitored for three years the number of papers submitted, unsubmitted for cell line issues, and resubmitted after uploading the appropriate proof of cell line authentication (Fig 3). In 2011, approximately 13% of papers received at the journal were unsubmitted for cell line issues and the authors were asked to upload the appropriate documents. In 2012 and 2013, the *IJC* enforced stricter rules and a higher percentage of papers (about 17%–18%) were unsubmitted. The majority of manuscripts unsubmitted were from Asia (more than 20% out of 1,200 submissions in 2012). In 2011, about half of the unsubmitted papers were resubmitted while the other half were withdrawn, and these numbers increased in 2012 and 2013.We analyzed whether there were differences in the resubmission rates between continents and found that a lower percentage of authors from Asia resubmitted their paper (49% compared to 71% and 64% for Europe and North America, respectively, in 2011–2013; Fig 4). It is possible that some authors who were nonnative speakers of English didn’t understand our requirements correctly or had problems uploading a certificate in English.

**Fig 5:**
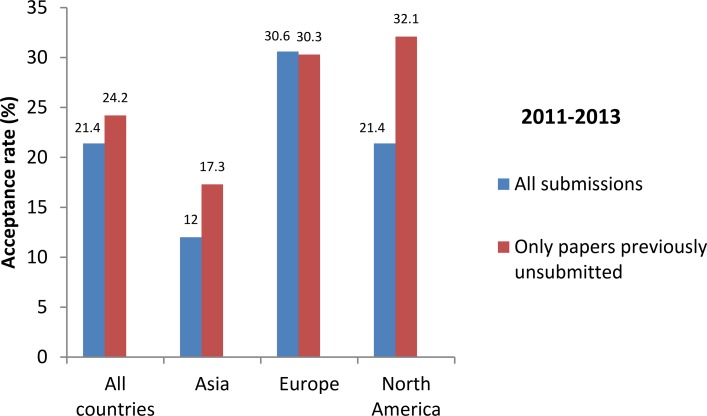
Acceptance rate for all submissions and for papers previously unsubmitted for cell line issues, by continent in 2011–2013. Generated from S1 Data, S2 Data, and S3 Data.

**Fig 6:**
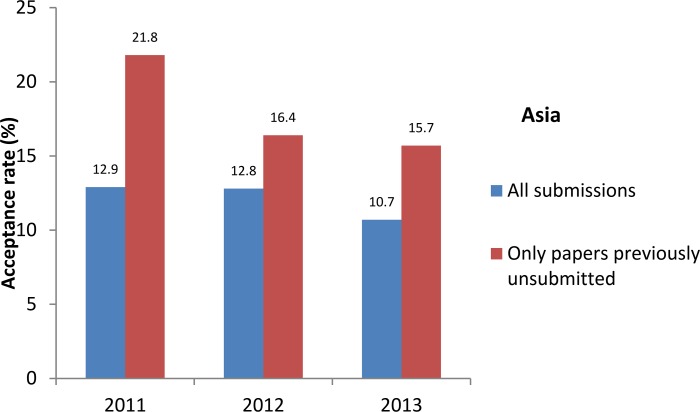
Acceptance rate for all submissions and for papers previously unsubmitted for cell line issues for Asia, by year. Generated from S1 Data, S2 Data, and S3 Data.

We also compared the acceptance rate for the papers previously unsubmitted with that for all papers received from 2011 to 2013 (Fig 5). There was a slight increase in the percentage of accepted papers for those previously unsubmitted, this difference being higher for papers from Asia and North America. This trend was relatively stable with time, as shown for Asia from 2011 to 2013 (Fig 6). These results show that the *IJC* didn’t lose papers worthy of acceptance; on the contrary, unsubmitting some papers seemed to introduce another element in our triage towards better submissions, and authors who wanted to publish in our journal were mainly not discouraged by such a requirement.

Thus, with easier authentication technologies and with more journals mandating cell line authentication before publication, acceptance and compliance with the new procedure have improved. An additional positive outcome was that our journal received a considerable amount of publicity, including reports in more widely read journals and magazines such as *Nature*, *BioTechniques*, and *Discover Magazine* as well as in the national press in Germany. In the long run, this publicity may have furthered this important effort and also enhanced the *IJC*’s reputation.

**Fig 7:**
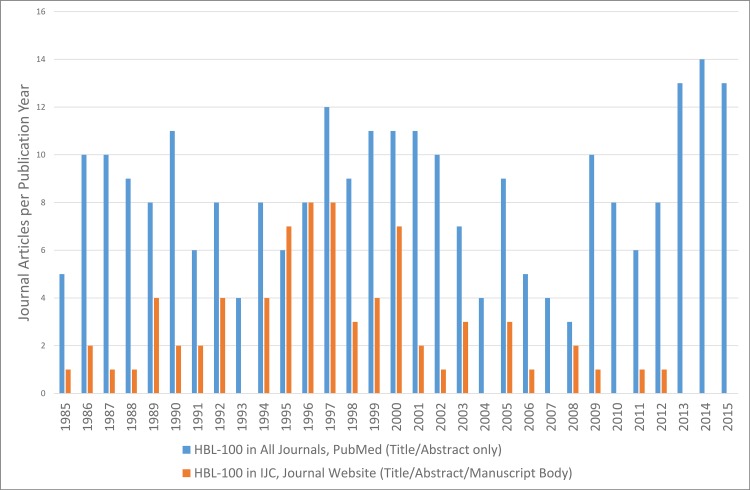
Journal articles using the misidentified cell line HBL-100.

It is important to ask whether these measures have addressed the problem of ongoing usage, however. For this, the PubMed searches shown in Fig 1 were examined to see how many of these journal articles were published in the *IJC*. From January 2010 to January 2016, using the misidentified cell line HBL-100, we were able to find only two articles that referred to this cell line in the title or abstract. Both were submitted in 2009, which was before the implementation of authentication requirements in 2011. This analysis also demonstrated the decrease in use of one such cell line, HBL-100, in the *IJC* since 2010 to zero, while it continued to increase dramatically in other PubMed-listed journals (Fig 7). It should be noted that PubMed searches give only an estimate of usage because they do not detect usage of misidentified cell lines in the body of the manuscript.

### Lessons learned

Ultimately, the hardest part of implementing the policy of requiring cell lines to be authenticated was taking the plunge and running the risk of not knowing how it would affect submissions, reputation, and workload for the editors and the editorial office. For the journal, it was a learning process during which procedures and processes evolved. For example, during this discussion, providing information about mycoplasma contamination was not considered obligatory for submission of a paper to the *IJC*, as it was thought to be taken care of by the investigators, just like the purity of any reagent used. However, as mycoplasma contamination has emerged as an increasing problem in published work, obligatory testing prior to submission will also be implemented at the *IJC* in the future. Another discussion point concerned limiting cell line documentation to a maximum of four years. This represented a compromise made for the initial phase of the process, but this will soon be substituted by a more stringent cutoff value. Needless to say, many intense discussions concerning these questions took place among the editors. Ultimately, we were all satisfied that our work had resulted in a drastic reduction of the use of misidentified cell lines in the *IJC* without a measurable loss in submissions but rather with a concomitantly increasing impact factor.

Consequently, we would recommend to other journals implementing such a requirement that everything be standardized at the outset: policy, procedures, and staffing. In the meantime, the goal is clear and the community is informed. Thus, it is only a matter of taking that step. Depending on an editorial office setup, it is more or less important for journals to save money or to add staff and relieve editors of some duties. For us, employing a dedicated person for that task proved to be the best choice: this has a monetary cost but brings benefits both in terms of educating young scientists who will possibly have their own laboratories in the future and also decreasing the editorial workload, particularly when editors are also still involved in their own research and do not work for the journal full-time. Finally, depending on the journal and rates of submissions from countries where English is less well understood, it might be useful to provide author instructions in other languages to meet potential communication problems head on.

Cell line authentication represents one of the quality control measures at our journal, and for the future, we will aim to further refine our policies here to include, for example, requiring a statement in the paper itself, extending the requirement to murine lines, and advertising to a broader readership that cell lines used in papers published in our journal have been authenticated. As we outlined in an editorial recently published in the journal [35], at the *IJC* we are aiming to apply similar standards to address the problem of antibody specificity and for describing the animals used in research in better detail. Also on this list are the issues of mycoplasma contamination and sharing and providing raw data.

## Conclusions

Up to now, only a few leading research laboratories and scientific journals are taking measures to change the dreadful situation of the continued use of wrongly denoted cell lines and, thereby, take responsibility for the publication of high-quality controlled data. Common excuses are that this would be the task of reviewers, scientific societies, and funding organizations. However, despite the fantastic work of individual reviewers, in general, the peer review process has mostly failed to detect and monitor the use of falsely designated cell lines. The still rising mountain of incorrect data can only be flattened by a concerted action of leading journal editors, research laboratories, cell banks, international scientific societies, and granting organizations. Beyond cell lines, adequate delineation of biological material poses a particular challenge for other tools and reagents as well, such as proteins or their modifications in tissue sections or fractionated samples using antibodies, patient-derived xenotransplants, or the detailed description of animals used in a study. The need to implement corresponding rules concerning information that is mandatory at submission to a journal has been recently discussed (see, e.g., [35]), and it is hoped that the lessons learned from implementing such a process for cell line authentication in the *International Journal of Cancer*—in particular, (i) providing exhaustive information to make the reasoning behind new rules transparent, (ii) meeting the arguments that are put forward as excuses for not following new quality measures, and (iii) shifting after a restricted period of discussion or voluntary following of rules to a strict algorithm—will promote this effort. Needless to say, corporate initiatives of journals and scientific societies would greatly foster such quality control measures and accelerate their broad acceptance, as would have happened if the *International Journal of Cancer* had found more fellow campaigners when the journal introduced mandatory cell line authentication. In addition, providing raw data and implementing rules for feeding data to be published in common databases, as pioneered by the community of genomics researchers, will constitute an important measure in support of these initiatives.

## Supporting information

S1 Data Supplementary data 1. (XLSX)

S2 Data Supplementary data 2. (XLSX)

S3 Data Supplementary data 3. (XLSX)

## Abbreviations

ATCC: American Type Culture Collection
IJC: International Journal of Cancer
ISMTE: International Society of Managing and Technical Editors
STR: short tandem repeat
